# Level of perception of technical terms regarding the effect of radiation on the human body by residents of Japan

**DOI:** 10.1186/s12199-017-0679-7

**Published:** 2017-10-27

**Authors:** Yoshitoku Yoshida, Yasuko Yoshida, Emiko Isogai, Takashi Hayase, Kozue Nakamura, Mitsuo Saito, Koji Arizono

**Affiliations:** 1 Faculty of Nursing, Shubun University, 6 Nikko-cho, Ichinomiya, Japan; 2 Graduate School of Medicine, Nagoya University, 65 Tsurumaicho, Showa-ku, Nagoya, 466-8550 Japan; 3 Graduate School of Pharmaceutical Sciences, Nagoya City University, 3-1 Tanabe-dori, Mizuho-ku, Nagoya, 467-8603 Japan; 4 Graduate School of Agricultural Science, Tohoku University, 468-1 Aoba, Aoba-ku, Sendai, Miyagi 980-0845 Japan; 5 Graduate School of Environmental Studies, Nagasaki University, Bunkyo-machi 1-14, Nagasaki, 852-8521 Japan; 6 Department of Food and Nutrition, Gifu City Women’s College, 7-1 Hitoichibakita-machi, Gifu, 501-0192 Japan; 7 Institute for Health Vigilance, 4-25-5-303, Tanashi-Cho, Nishi-Tokyo, Tokyo 188-0011 Japan; 8 Faculty of Environmental Symbiotic Science, Prefectural University of Kumamoto, Tsukide 3-1-100, Higashi-ku, Kumamoto, 862-8502 Japan

**Keywords:** Risk communication, Perception gap, Great East Japan Earthquake, Radiation, Reliability

## Abstract

**Background:**

This study aimed to examine the level of perception of the technical terms related to the effect of radiation on the human body among residents of the six prefectures of Miyagi, Fukushima, Tokyo, Aichi, Hiroshima, and Nagasaki in Japan. Miyagi and Fukushima were selected as devastated area by Great East Japan Earthquake. Tokyo and Aichi were selected as control. Hiroshima and Nagasaki were selected as the A-bombed area.

**Methods:**

A total of 1030 respondents, 172, 173, 171, 173, 171, and 170, respectively, were surveyed. Differences in the recognition level of technical terms related to the effect of radiation on the human body among residents of the six prefectures were assessed.

**Results:**

The highest recognition levels were reported by the respondents from Fukushima (17 items). Those from Miyagi scored the second highest recognition levels (10 out of the 17 terms); the second highest recognition levels for the remaining seven terms were marked by the respondents of Tokyo. Respondents in the Tohoku region had a better recognition for the technical terminology relevant to the effect of radiation on the human body.

**Conclusions:**

Our findings indicate a need for continued, comprehensive risk communication pertaining to health hazards of radiation exposure in Tohoku region. Concerted efforts by central/local governments and other stakeholders are required to allay the anxiety/stress related to radiation exposure among the residents.

## Background

On March 11, 2011, a devastating earthquake, tsunami, and subsequent Fukushima nuclear power plant accident occurred in Japan. According to Ministry of International Affairs and Communications of Japan, a total of 22,010 people died or were reported as missing after the Great East Japan Earthquake. Further, a total of 400,305 buildings were destroyed in the wake of the disaster [1]. At its peak, the number of refugees in the aftermath of the disaster was approximately 470,000; as of December 10, 2015, the number of refugees stands at 182,000 [2, 3]. Wild mushrooms were collected in Kawauchi Village after the accident at the Fukushima Daiichi Nuclear Power Plant. The radio-cesium concentrations of them were calculated to be committed effective doses. However, Nakashima et al. mentioned that the committed effective doses were limited even if residents ate contaminated foods several times and that the comprehensive risk communication based on the results of the radio-cesium measurements of food, water, and soil was necessary for the recovery of Fukushima after this nuclear disaster [4]. Although some parts of the Kanto area continue to show elevated environmental radiation levels, the ambient dose rate and radioactive contamination of foodstuffs produced inside the city have been monitored [5]. Iimoto et al. reported that representative ambient dose rates around residential areas have almost returned to the original pre-accident levels because of the decontamination activities, natural washout, and the low effective half-lives of radioactivity [6]. In 2003, the Food Safety Basic Law was established in Japan to address food safety issues such as bovine spongiform encephalopathy. This law stipulates that the central government should conduct risk communication to ensure food safety and to reassure the nation. Therefore, forum-style risk communication in the field of food safety and environmental conservation practiced by stakeholders such as the government and citizen representatives have been conducted vigorously even though still now there are much room for improvement [7, 8]. Providing precise information about the risks and promoting the exchange of opinions between stakeholders are essential to tackle the facing difficulties.

In the Tohoku region, to reduce the anxiety and mental stress related to radiation exposure among evacuees, extensive interventions for risk communication about radiation exposure to the human body were implemented by various stakeholders, including the central and local governments. In a previous study, workers and mothers of young children were shown to be at a higher risk of depression, anxiety, and psychosomatic and post-traumatic disorders, both as a direct result of their fears about radiation exposure and as an indirect result of societal stigma [9, 10]. The importance of risk communication in the devastated area has been highlighted in several reports [4, 11, 12]. However, an experts’ perception of the risk of exposure to medical X-rays and natural sources of radiation is significantly higher than that of the general population, while experts have lower risk perception of the radiation hazard posed by nuclear waste or accident at a nuclear installation, as compared to that of general population [13]. In addition, we have earlier demonstrated the perception gaps related to pharmaceutical terms and related issues between the lay population and medical doctors [14], pharmacists [15], and nurses [16]. To conduct risk communications in the devastated area smoothly and effectively, interventions for risk communication in areas affected by disasters should be preceded by efficacy evaluation of the methodology used. Therefore, the purpose of this study was to evaluate the effectiveness of risk communications with the recognition level of technical terms relevant to the radiation exposure to the human body.

## Methods

### Study population and design

Demographic data pertaining to residents of six prefectures, such as age, gender, education, and income, were collected in this study. The residents from six prefectures (Miyagi, Fukushima, Tokyo, Aichi, Hiroshima, and Nagasaki) were examined as the subjects of this study. Miyagi and Fukushima were selected as devastated area by Great East Japan Earthquake. Tokyo and Aichi were selected as a control. Hiroshima and Nagasaki were selected as the A-bombed area.

### Data collection

The research was performed under contract with NTT Rezonanto Co., Ltd., using an Internet survey monitored by Goo Research contractors. The study was conducted from February 13 to 19, 2014. Based on the results of the similar researches [14–16], a sample size of 160 was determined for each prefecture. In details, the research was conducted in line with the rules of NTT Rezonanto Co., Ltd., as follows: samples representing 110% of sample size of 160 were collected from the monitors whose demographics were preregistered, and the first 3–5% of respondents who answered the questions in a very short period were evaluated as imperfect and submitted to us after removing the imperfect samples.

Monitors from six prefectures have been previously registered with an Internet research company. The basic information such as gender and residence of the monitors has been registered as well. As an incentive, each monitor is awarded 50 points (about 50 cents) after completing the answers to the questions.

### Questionnaires and statistical analyses

Questionnaires consisted of demographic characteristics of respondents, number of participations to the forum/meeting of the risk communication relating to the effect of radiation to the human body, awareness of the recognition level of the 20 technical terms (as shown in Table 5) regarding radiation, awareness of the recognition level of the meanings of eight technical issues (as shown in Table 6) regarding radiation, necessity on the knowledge which should be delivered at the forum-style risk communication, the sources of information regarding radiation and its reliability among residents of the six prefectures and the reliability of central government, and risk avoidance behavior regarding radiation.

In many cases of using 5 Likert scale, to make definition of number clear, usually, 1, 3, and 5 were explained explicitly, but 2 and 4 were explained implicitly [14–16].

The recognition level of the 20 technical terms regarding radiation among residents of the six prefectures, the residents were required to indicate their responses on a 5-point Likert scale: 1: “I do not see or hear it”; 3: “I cannot tell clearly whether I see or hear it”; and 5: “I often see or hear it.” The most appropriate number between 1 and 5 was selected. The variable was termed as “the recognition level of the technical term regarding radiation.” For data analysis, responses 4 and 5 were merged under the category as “I know.”

For assessment of the recognition level for eight technical issues related to radiation and the knowledge which should be delivered at the forum-style risk communication among residents of the six prefectures, the response options on the 5-point Likert scale were 1: “I do not know it,” 3: “I cannot tell clearly whether I know it,” and 5: “I know it very well.” The most appropriate number between 1 and 5 was selected by the respondents. The variable was termed as “the recognition level of the meaning of the technical issue related to radiation.” For data analysis, responses 4 and 5 were merged under the category as “I know.”

In terms of the differences in the knowledge which should be delivered in a forum-style risk communication session among residents of the six prefectures, the response options were 1: “I do not need it,” 3: “I cannot tell clearly whether I need it,” and 5: “I need it very well.” The most appropriate number between 1 and 5 was selected. The variable was termed as “knowledge which should be delivered at the forum-style risk communication.” For data analysis, response options 4 and 5 were merged under the category as “I need.”

In terms of the sources of information related to radiation and with respect to the reliability of central government sources and radiation risk avoidance behavior, the response options were 1: “I do not agree with it,” 3: “I cannot tell clearly whether I agree on it,” and 5: “I agree with it very well.” The most appropriate number between 1 and 5 was selected. It was termed “sources of information regarding radiation and its perceived reliability and the reliability of the central government and risk avoidance behavior related to radiation.”

For the purpose of analysis, 4 and 5 were merged under the category as “I agree.” Chi-square test and binary logistic regression were employed for data analysis.

## Results

Table 1 summarizes the demographic characteristics of the respondents. A statistically significant difference was observed between respondents in the six prefectures with respect to sex distribution, education level, and income of the respondents. Females accounted for the majority of respondents in Nagasaki (55.9%), while males were in majority in the other five prefectures (Miyagi [51.7%], Fukushima [61.8%], Tokyo [54.4%], Aichi [54.3%], Hiroshima [52.6%]). The number of respondents of Nagasaki who graduated high school occupied the most (37.6%), whereas the number of respondents of the other five prefectures, Miyagi, Fukushima, Tokyo, Aichi, and Hiroshima, who graduated in a university, were the highest proportion (37.8, 35.3, 57.3, 43.4, and 48.0%), respectively. The number of respondents of Miyagi and Nagasaki whose income was 2,000,000 JPY–4000,000 JPY showed the majority (27.9 and 36.5%), respectively, whereas the number of respondents of Fukushima, Tokyo, Aichi, and Hiroshima whose income was 4000,000 JPY–6000,000 JPY were the highest proportion (25.4, 26.3, 29.5, and 28.7%), respectively. However, there was no statistically significant difference in age between respondents from the six prefectures.

**Table 1:** Demographic characteristics of respondents

		Residence							
		Miyagi (*n* = 172)	Fukushima (*n* = 173)	Tokyo (*n* = 171)	Aichi (*n* = 173)	Hiroshima (*n* = 171)	Nagasaki (*n* = 170)	Total (*n* = 1030)	Test
Sex	Male	89(51.7%)	107(61.8%)	93(54.4%)	94(54.3%)	90(52.6%)	75(44.1%)	548(53.2%)	*
	Female	83(48.3%)	66(38.2%)	78(45.6%)	79(45.7%)	81(47.4%)	95(55.9%)	482(46.8%)	
Age (years)	20–29	13(7.6%)	7(4.0%)	20(11.7%)	17(9.8%)	20(11.7%)	9(5.3%)	86(8.3%)	n.s.
	30–39	38(22.1%)	38(22.0%)	27(15.8%)	38(22.0%)	41(24.0%)	31(18.2%)	213(20.7%)	
	40–49	57(33.1%)	58(33.5%)	48(28.1%)	60(34.7%)	52(30.4%)	55(32.4%)	330(32.0%)	
	50–59	42(24.4%)	42(24.3%)	50(29.2%)	40(23.1%)	35(20.5%)	46(27.1%)	255(24.8%)	
	60–69	18(10.5%)	19(11.0%)	18(10.5%)	13(7.5%)	18(10.5%)	25(14.7%)	111(10.8%)	
	≥ 70	4(2.3%)	9(5.2%)	8(4.7%)	5(2.9%)	5(2.9%)	4(2.4%)	35(3.4%)	
Education level	Junior high school	4(2.3%)	6(3.5%)	0(0.0%)	6(3.5%)	1(0.6%)	4(2.4%)	21(2.0%)	**
	High school	63(36.6%)	58(33.5%)	31(18.1%)	52(30.1%)	42(24.6%)	64(37.6%)	310(30.1%)	
	College/vocational school	31(18.0%)	42(24.3%)	35(20.5%)	32(18.5%)	33(19.3%)	45(26.5%)	218(21.2%)	
	University	65(37.8%)	61(35.3%)	98(57.3%)	75(43.4%)	82(48.0%)	50(29.4%)	431(41.8%)	
	Graduate school	9(5.2%)	6(3.5%)	7(4.1%)	8(4.6%)	13(7.6%)	7(4.1%)	50(4.9%)	
Income (JPY)	Less than 2,000,000	21(12.2%)	32(18.5%)	13(7.6%)	16(9.2%)	21(12.3%)	20(11.8%)	123(11.9%)	**
	2,000,000–4000,000	48(27.9%)	39(22.5%)	36(21.1%)	40(23.1%)	48(28.1%)	62(36.5%)	273(26.5%)	
	4000,000–6000,000	44(25.6%)	44(25.4%)	45(26.3%)	51(29.5%)	49(28.7%)	49(28.8%)	282(27.4%)	
	6000,000–8,000,000	41(23.8%)	32(18.5%)	33(19.3%)	30(17.3%)	31(18.1%)	19(11.2%)	186(18.1%)	
	≥ 8,000,000	18(10.5%)	26(15.0%)	44(25.7%)	36(20.8%)	22(12.9%)	20(11.8%)	166(16.1%)	

*χ* ^2^ test

*JPY* Japanese yen, *n.s.* not significant

**p < 0.01

Table 2 shows the number of participants in meetings for risk communication related to the effect of radiation on the human body. Prior to the East Japan Great Earthquake, there was no significant difference in the level of participation in the sessions for risk communication related to the effect of radiation on the human body. However, after the earthquake, there was a significant difference in the level of participation between the six prefectures. In Fukushima, 11.0% of respondents had participated in the meeting once and 11.0% of respondents had participated in the meeting on two or more occasions.

**Table 2:** Number of participations to the forum/meeting of the risk communication relating to the effect of radiation to the human body

	Before the Great East Japan Earthquake								After the Great East Japan Earthquake							
	0		1		≥ 2		Total	Test	0		1		≥ 2		Total	Test
Miyagi	162	94.2%	8	4.7%	2	1.2%	172	n.s.	160	93.0%	6	3.5%	6	3.5%	172	**
Fukushima	167	96.5%	1	0.6%	5	2.9%	173		135	78.0%	19	11.0%	19	11.0%	173	
Tokyo	167	97.7%	2	1.2%	2	1.2%	171		166	97.1%	2	1.2%	3	1.8%	171	
Aichi	171	98.8%	1	0.6%	1	0.6%	173		167	96.5%	4	2.3%	2	1.2%	173	
Hiroshima	164	95.9%	3	1.8%	4	2.3%	171		166	97.1%	3	1.8%	2	1.2%	171	
Nagasaki	161	94.7%	5	2.9%	4	2.4%	170		164	96.5%	3	1.8%	3	1.8%	170	

*χ* ^2^ test

*n.s.* not significant

**p < 0.01

Factors associated with the recognition level for the 20 technical terms related to radiation were analyzed by binary logistic regression model to assess the crude odds ratio (OR) and adjusted OR. The individual scores for recognition level for each of the 20 technical terms were added to obtain a total score. In the case of using 5 Likert scale, to make two groups, score 3 of the median is commonly adopted as a cut-off level. Therefore, a cut-off level of 61 (3 multiplied by 20 plus 1) was adopted to classify the recognition level of the respondent; a total score of ≥ 61 was defined as “know.” In the unadjusted analysis, statistically significant associations of “know” were observed on sub-group analysis (Table 3). Among those who were male, those in the age groups of 40–49 years, 50–59 years, and 60–69 years, and those living in Miyagi and Fukushima were more likely to “know,” compared to those who were female, 20–29 years old, and living in Aichi (OR = 2.266, 95%CI 1.675–3.065, *p* = 0.000; OR = 1.920, 95%CI 1.147–3.212, *p* = 0.013; OR = 2.371, 95%CI 1.375–4.088, *p* = 0.002; OR = 2.437, 95%CI 1.264–4.699, *p* = 0.008; OR = 2.372, 95%CI 1.406–4.001, *p* = 0.001; OR = 4.334, 95%CI 2.361–7.956, *p* = 0.000), respectively. After adjusting for sex, age, education, income, and residence, the demographic factors that showed significant association with “know” were sex (adjusted OR = 1.858; 95%CI 1.212–2.631; *p* = 0.000, for male versus female) and age (adjusted OR = 1.827; 95%CI 1.011–3.300; *p* = 0.046, for 50–59 years old versus 20–29 years). Residence was also significantly associated with “know” (adjusted OR = 2.693, 95%CI 1.570–4.621, *p* = 0.000; adjusted OR = 4.627, 95%CI 2.479–8.635, *p* = 0.000, for living in Miyagi and Fukushima versus living in Aichi, respectively). Factors associated with the recognition level of the meanings of eight technical issues related to radiation were analyzed using a binary logistic regression model to assess the crude OR and adjusted OR. The individual scores for recognition level for the eight technical issues related to radiation were added to obtain a total score. A cut-off level of 25 (3 multiplied by 8 plus 1) was adopted to determine the recognition level of a respondent; a total score ≥ 25 was defined as “know.”

**Table 3:** Odds ratios (OR) and 95% confidence intervals (CI) for awareness of the recognition level of the 20 technical terms regarding radiation

		Know^a^ *N* (%)		Crude OR (95% CI)^b^	*p*	Adjusted OR (95% CI)^c^	*p*
Sex	Female	339/482	(70.3)	1 (reference)		1 (reference)	
	Male	462/548	(84.3)	2.266 (1.675–3.065)	< 0.000	1.858 (1.312–2.631)	< 0.000
Age	20–29	56/86	(65.1)	1 (reference)		1 (reference)	
	30–39	162/213	(76.1)	1.702 (0.988–2.931)	0.055	1.452 (0.819–2.575)	0.202
	40–49	258/330	(78.2)	1.920 (1.147–3.212)	0.013	1.625 (0.935–2.826)	0.085
	50–59	208/255	(81.6)	2.371 (1.375–4.088)	0.002	1.827 (1.011–3.300)	0.046
	60–69	91/111	(82.0)	2.437 (1.264–4.699)	0.008	1.674 (0.820–3.417)	0.157
	≥ 70	26/35	(74.3)	1.548 (0.643–3.724)	0.330	1.111 (0.432–2.858)	0.827
Education	Junior high school	15/21	(71.4)	1 (reference)		1 (reference)	
	High school	225/310	(72.6)	1.059 (0.398–2.819)	0.909	1.079 (0.377–3.089)	0.887
	College/vocational school	159/218	(72.9)	1.078 (0.399–2.909)	0.882	1.334 (0.457–3.893)	0.598
	University	358/431	(83.1)	1.962 (0.736–5.225)	0.178	2.017 (0.701–5.798)	0.193
	Graduate school	44/50	(88.0)	2.933 (0.820–10.490)	0.098	3.071 (0.795–11.858)	0.104
Income (JPY)	< 2,000,000	94/123	(76.4)	1 (reference)		1 (reference)	
	2,000,000–4000,000	207/273	(75.8)	0.968 (0.587–1.595)	0.897	1.064 (0.625–1.811)	0.819
	4000,000–6000,000	219/282	(77.7)	1.072 (0.649–1.771)	0.785	1.116 (0.653–1.906)	0.688
	6000,000–8,000,000	141/186	(75.8)	0.967 (0.566–1.650)	0.901	0.790 (0.442–1.411)	0.426
	> 8,000,000	140/166	(84.3)	1.661 (0.921–2.998)	0.092	1.411 (0.745–2.672)	0.291
Residence	Aichi	120/173	(69.4)	1 (reference)		1 (reference)	
	Nagasaki	121/170	(71.2)	1.091 (0.686–1.733)	0.714	1.227 (0.755–1.996)	0.409
	Hiroshima	127/171	(74.3)	1.275 (0.796–2.042)	0.313	1.296 (0.793–2.117)	0.300
	Tokyo	131/171	(76.6)	1.446 (0.896–2.336)	0.131	1.358 (0.823–2.241)	0.231
	Miyagi	145/172	(84.3)	2.372 (1.406–4.001)	0.001	2.693 (1.570–4.621)	< 0.000
	Fukushima	157/173	(90.8)	4.334 (2.361–7.956)	< 0.000	4.627 (2.479–8.635)	< 0.000

*N* number of subjects

^a^1 means “I do not see or hear it.” 3 means “I cannot tell clearly whether I see or hear it”, 5 means “I often see or hear it.” In analyzing, 4 and 5 out of 1 to 5 were used as “I know”

^b^Crude odds ratio and 95% confidence interval

^c^Adjusted odds ratio for sex, age, education, income, residence, and 95% confidence interval

In the unadjusted analysis, there was a statistically significant association of “know” as shown in Table 4. Among those who were male, 40–49 years old, 50–59 years old, and living in Nagasaki, Hiroshima, Tokyo, Miyagi, and Fukushima were more likely to “know,” compared to those who were female, 20–29 years old, and lived in Aichi (OR = 1.434, 95%CI 1.113–1.848, *p* = 0.005; OR = 1.834, 95%CI 1.091–3.083, *p* = 0.022; OR = 1.751, 95%CI 1.027–2.984, *p* = 0.040; OR = 1.840, 95%CI 1.139–2.972, *p* = 0.013; OR = 2.179, 95%CI 1.356–3.500, *p* = 0.001; OR = 1.920, 95%CI 1.191–3.096, *p* = 0.007; OR = 2.267, 95%CI 1.413–3.637, *p* = 0.001; OR = 6.051, 95%CI 3.766–9.721, *p* = 0.000), respectively. After adjusting for sex, age, education, income, and residence, only residence was associated with a significant association with “know” (adjusted OR = 1.977, 95%CI 1.207–3.237, *p* = 0.007; adjusted OR = 2.171, 95%CI 1.337–3.525, *p* = 0.002; adjusted OR = 1.903, 95%CI 1.168–3.102, *p* = 0.010; adjusted OR = 2.326, 95%CI 1.436–3.770, *p* = 0.001; adjusted OR = 6.492, 95%CI 3.994–10.551, *p* = 0.000, for living in Nagasaki, Hiroshima, Tokyo, Miyagi, and Fukushima versus living in Aichi, respectively).

**Table 4:** Odds ratio (OR) and 95% confidence interval (CI) for awareness of the recognition level of the meanings of eight technical issues regarding radiation

		Know^a^/*N* (%)		Crude OR (95% CI)^b^	*p*	Adjusted OR (95% CI)^c^	*p*
Sex	Female	164/482	(34.0)	1 (reference)		1 (reference)	
	Male	233/548	(42.5)	1.434 (1.113–1.848)	0.005	1.175(0.869–1.589)	0.295
Age	20–29	24/86	(27.9)	1 (reference)		1 (reference)	
	30–39	77/213	(36.2)	1.463 (0.846–2.530)	0.174	1.230 (0.692–2.186)	0.480
	40–49	137/330	(41.5)	1.834 (1.091–3.083)	0.022	1.576 (0.906–2.742)	0.108
	50–59	103/255	(40.4)	1.751 (1.027–2.984)	0.040	1.485 (0.834–2.642)	0.179
	60–69	44/111	(39.6)	1.697 (0.926–3.108)	0.087	1.450 (0.752–2.795)	0.268
	≥ 70	12/35	(34.3)	1.348 (0.581–3.129)	0.487	1.071 (0.435–2.636)	0.881
Education	Junior high school	8/21	(38.1)	1 (reference)		1 (reference)	
	High school	108/310	(34.8)	0.869 (0.349–2.161)	0.762	0.833 (0.313–2.212)	0.713
	College/vocational school	77/218	(35.3)	0.887 (0.352–2.235)	0.800	0.874 (0.322–2.369)	0.791
	University	172/431	(39.9)	1.079 (0.438–2.658)	0.868	1.083 (0.409–2.864)	0.873
	Graduate school	32/50	(64.0)	2.889 (1.008–8.282)	0.048	3.083 (0.997–9.541)	0.051
Income (JPY)	Less than 2,000,000	44/123	(35.8)	1 (reference)		1 (reference)	
	2,000,000–4000,000	98/273	(35.9)	1.005 (0.645–1.567)	0.981	1.163 (0.724–1.868)	0.532
	4000,000–6000,000	106/282	(37.6)	1.081 (0.696–1.680)	0.728	1.198 (0.747–1.922)	0.454
	6000,000–8,000,000	81/186	(43.5)	1.385 (0.867–2.214)	0.173	1.373 (0.824–2.286)	0.223
	> 8,000,000	68/166	(41.0)	1.246 (0.770–2.016)	0.371	1.238 (0.729–2.103)	0.429
Residence	Aichi	38/173	(22.0)	1 (reference)		1 (reference)	
	Nagasaki	58/170	(34.1)	1.840 (1.139–2.972)	0.013	1.977 (1.207–3.237)	0.007
	Hiroshima	65/171	(38.0)	2.179 (1.356–3.500)	0.001	2.171 (1.337–3.525)	0.002
	Tokyo	60/171	(35.1)	1.920 (1.191–3.096)	0.007	1.903 (1.168–3.102)	0.010
	Miyagi	67/172	(39.0)	2.267 (1.413–3.637)	0.001	2.326 (1.436–3.770)	0.001
	Fukushima	109/173	(63.0)	6.051 (3.766–9.721)	< 0.000	6.492(3.994–10.551)	< 0.000

*N* the number of subjects for the category

^a^The score of the recognition level of the 20 technical terms regarding radiation was summed, and 61 was adopted as the cut-off level. This means 61 or more of the total recognition level of the 20 technical terms regarding radiation were defined as “know”

^b^Crude odds ratio and 95% confidence interval

^c^Adjusted odds ratio for sex, age, education, income, residence, and 95% confidence interval

Overall, 89.2% of the respondents had knowledge of radioactive substances (Table 5). Prevalence of knowledge related to the individual terms were Sievert (88.2%), internal radiation exposure (86.7%), radioactive cesium (86.2%), WHO (World Health Organization) (85.7%), X-ray (84.4%), Becquerel (83.4%), external radiation exposure (80.8%), IAEA (International Atomic Energy Agency) (71.0%), radioactive strontium (61.5%), natural background radiation (60.0%), half-life (58.6%), β-ray (53.2%), low-dose radiation exposure (52.8%), neutron ray (50.9%), α-ray (47.8%), γ-ray (46.0%), acute radiation exposure (45.1%), ICRP (International Commission on Radiological Protection) (25.5%), and the law concerning the prevention from radiation hazards due to radioisotopes and others of Japan (19.3%) (Table 5).

**Table 5:** Difference in the percentage of “Know” on the recognition level of the 20 technical terms regarding radiation among residents of the six prefectures

	Miyagi (*n* = 172)	Fukushima (*n* = 173)	Tokyo (*n* = 171)	Aichi (*n* = 173)	Hiroshima (*n* = 171)	Nagasaki (*n* = 170)	Total (*n* = 1030)	
Term	Know (%)	Know (%)	Know (%)	Know (%)	Know (%)	Know (%)	Know (%)	Test
Radioactive substance	94.8	95.4	89.5	85.0	86.0	84.7	89.2	**
Sievert	94.2	95.4	87.1	84.4	83.6	84.1	88.2	**
Internal radiation exposure	91.3	96.0	87.1	80.3	83.0	82.4	86.7	**
Radioactive cesium	90.7	95.4	85.4	82.7	80.1	82.9	86.2	**
WHO (World Health Organization)	84.3	87.3	88.9	86.1	81.9	85.9	85.7	n.s.
X-ray	86.0	89.0	81.9	82.7	80.1	86.5	84.4	n.s.
Becquerel	91.3	94.8	80.1	77.5	76.6	80.0	83.4	**
External radiation exposure	84.3	93.6	81.3	71.7	76.0	77.6	80.8	**
IAEA (International Atomic Energy Agency)	70.9	83.8	77.2	65.3	63.7	64.7	71.0	**
Radioactive strontium	64.0	85.0	63.2	49.7	54.4	52.4	61.5	**
Natural background radiation	59.9	80.9	61.4	47.4	55.0	55.3	60.0	**
Half-life	59.3	86.7	60.8	51.4	50.9	42.4	58.6	**
β-ray	54.7	67.6	54.4	42.8	48.5	51.2	53.2	**
Low-dose radiation exposure	53.5	78.6	52.0	35.8	50.3	46.5	52.8	**
Neutron ray	44.2	64.7	51.5	48.0	47.4	49.4	50.9	**
α-ray	49.4	60.1	48.0	39.9	44.4	44.7	47.8	**
γ-ray	43.6	58.4	50.9	41.6	39.8	41.8	46.0	**
Acute radiation exposure	44.2	60.1	48.0	32.9	43.3	42.4	45.1	**
ICRP (International Commission on Radiological Protection)	21.5	41.6	25.7	17.3	24.0	22.9	25.5	**
The law concerning the prevention from radiation hazards due to radioisotopes and others of Japan	16.9	27.2	18.7	13.9	19.3	20.0	19.3	n.s.

*χ* ^2^ test

*n.s.* not significant

**p < 0.01

Regarding the recognition level for the 20 technical terms related to radiation, the highest recognition level was among the respondents in Fukushima (17 terms). The second highest recognition level of the 10 terms out of 17 was among the respondents in Miyagi, and the second highest recognition levels of the remaining seven terms were marked by the respondents of Tokyo. The three terms for which no statistically significant differences in recognition level were observed among the six prefectures were WHO, X-ray, and the Radiation Hazards Prevention Law in Japan.

Overall, 38.2% of the total respondents were aware of the exposure dosage from natural background radiation (Table 6). The results included exposure dosage by everyday life radiation (37.4%), relation between radiation exposure and health effects (35.8%), relation between radiation exposure and disease (34.2%), existence of diseases susceptible to radiation exposure (30.4%), many of the international standards on the health effects of radiation are estimated (29.9%), differences between Becquerel and Sievert (23.6%), and the existence of a threshold of radiation exposure to health effects (16.1%). The highest recognition level for all eight issues was indicated by the respondents of Fukushima. The second highest recognition levels were the five technical issues out of the eight marked by the respondents of Miyagi, and the recognition levels for two technical issues and one technical issue were marked by the respondents of Hiroshima and Nagasaki, respectively.

**Table 6:** Difference in the percentage of “Know” on the recognition level of the meanings of eight technical issues regarding radiation among residents of the six prefectures

	Miyagi (*n* = 172)	Fukushima (*n* = 173)	Tokyo (*n* = 171)	Aichi (*n* = 173)	Hiroshima (*n* = 171)	Nagasaki (*n* = 170)	Total (*n* = 1030)	
Item	Know (%)	Know (%)	Know (%)	Know (%)	Know (%)	Know (%)	Know (%)	Test
Exposure dosage by natural background radiation	39.0	65.3	34.5	22.0	33.9	34.1	38.2	**
Exposure dosage by everyday life radiation	40.1	61.3	35.1	21.4	32.7	33.5	37.4	**
Relation between radiation exposure and health effects	31.4	48.6	36.8	22.0	43.9	32.4	35.8	**
Relation between radiation exposure and diseases	34.3	46.8	29.2	23.7	36.8	34.1	34.2	**
Existence of diseases susceptible to radiation exposure	29.7	42.2	26.3	20.8	31.6	31.8	30.4	**
Many of the international standards on the health effects of radiation are estimated	34.9	48.6	29.2	16.2	27.5	22.9	29.9	**
Difference between Becquerel and Sievert	29.7	43.4	24.0	10.4	18.7	15.3	23.6	**
Existence of threshold of radiation exposure to health effects	19.2	24.9	15.8	8.7	15.2	12.9	16.1	**

*χ* ^2^ test

*n.s.* not significant

**p < 0.01

Table 7 demonstrates the difference in the percentage of respondents “Need” on the knowledge, which should be delivered at the forum-style risk communication. Overall, 74.7% of the total respondents were aware of the risk of radiation exposure. We found the following data: the effectiveness of the food standards as a radiation countermeasure (73.3%), scientific facts (72.4%), radiation effects on children (71.6%), setting the basis for the standards (69.3%), the effectiveness of decontamination as a radiation countermeasure (68.3%), validity of the standards (68.1%), the effectiveness of the standards of space dose of radiation as a countermeasure (66.4%), recommendation of avoidance behavior (65.0%), and the uncertainty of radiation effects (59.5%). Based on the differences in the knowledge that should be delivered at a forum-style risk communication, the highest values of percentage of the four terms out of seven were marked by the respondents of Fukushima. The next highest were marked by the respondents of Miyagi. The highest values of percentage of the remaining three were marked by the respondents of Miyagi; the next were marked by the respondents of Fukushima.

**Table 7:** Difference in the percentage of “Need” on the knowledge which should be delivered at the forum-style risk communication among residents of the six prefectures

	Miyagi (*n* = 172)	Fukushima (*n* = 173)	Tokyo (*n* = 171)	Aichi (*n* = 173)	Hiroshima (*n* = 171)	Nagasaki (*n* = 170)	Total (*n* = 1030)	
Item	Need (%)	Need (%)	Need (%)	Need (%)	Need (%)	Need (%)	Need (%)	Test
Risk of radiation effects	79.1	82.7	71.9	72.3	69.6	72.4	74.7	*
Effectiveness of food standards as a radiation countermeasure	79.7	78.0	71.9	72.8	66.1	71.2	73.3	n.s.
Scientific facts	80.8	75.7	74.3	68.2	64.9	70.6	72.4	*
Radiation effects on children	79.7	74.0	68.4	69.4	64.3	73.5	71.6	*
Setting the basis for the standards	76.7	77.5	68.4	68.2	60.2	64.7	69.3	**
Effectiveness of decontamination as a radiation countermeasure	73.3	73.4	67.3	65.3	62.6	67.6	68.3	n.s.
Validity of the standards	78.5	75.1	66.7	67.1	60.2	60.6	68.1	**
The effectiveness of the standards of space dose of radiation as a countermeasure	70.9	77.5	64.9	63.6	56.7	64.7	66.4	**
Recommendation of avoidance behavior	68.6	69.9	63.7	65.3	61.4	61.2	65.0	n.s.
Uncertainty of radiation effects	64.5	71.1	57.3	53.8	56.7	53.5	59.5	**

*χ* ^2^ test

*n.s.* not significant

**p < 0.01; *p < 0.05

Table 8 demonstrates the difference in the percentage of “Agree” on the sources of information regarding radiation and its reliability. Overall, 57.5% of respondents consider TV to be a resource. The following data were determined for these sources: Internet (53.6%), newspaper (52.8%), public relations exercise by the local government (37.9%), public relations by the central government (33.7%), forum/meeting of risk communication (31.4%), academic journals/technical books (29.7%), and radio (25.9%). There were no statistically significant differences in the perceived reliability of the eight sources of information between respondents in the six prefectures (Table 8). Overall, 51.2% of respondents perceived the academic journals/technical books to be a reliable source of information. The corresponding data for other sources of information were forum/meeting for risk communication (42.0%), newspapers (41.3%), public relations by the local government (38.6%), TV (34.5%), public relations by the central government (34.4%), Internet (29.3%), and radio (26.2%).

**Table 8:** Difference in the percentage of “Agree” on the sources of information regarding radiation and its reliability among residents of the six prefectures

	Miyagi (*n* = 172)	Fukushima (*n* = 173)	Tokyo (*n* = 171)	Aichi (*n* = 173)	Hiroshima (*n* = 171)	Nagasaki (*n* = 170)	Total (*n* = 1030)	
Source	Agree (%)	Agree (%)	Agree (%)	Agree (%)	Agree (%)	Agree (%)	Agree (%)	Test
TV	55.2	55.5	57.3	58.4	59.6	58.8	57.5	n.s.
Internet	45.3	54.3	57.9	54.3	56.1	53.5	53.6	n.s.
Newspaper	53.5	52.6	54.4	52.0	53.2	51.2	52.8	n.s.
The public relations by the local government	39.5	42.2	40.9	35.8	27.5	41.2	37.9	*
The public relations by central government	28.5	34.1	40.4	34.7	26.3	38.2	33.7	*
Forum/meeting of risk communication	32.0	39.3	25.7	31.8	25.7	33.5	31.4	n.s.
Academic journals/technical book	30.8	31.2	31.6	27.7	22.8	34.1	29.7	n.s.
Radio	25.6	28.9	28.1	27.2	22.2	23.5	25.9	n.s.
Reliability	Agree (%)	Agree (%)	Agree (%)	Agree (%)	Agree (%)	Agree (%)	Agree (%)	test
Academic journals/technical book	52.9	41.6	54.4	50.9	52.0	55.3	51.2	n.s.
Forum/meeting of risk communication	43.0	38.7	39.8	39.9	43.9	47.1	42.0	n.s.
Newspaper	43.0	33.5	39.8	45.7	40.4	45.3	41.3	n.s.
The public relations by the local government	40.7	32.9	34.5	37.6	39.2	47.1	38.6	n.s.
TV	33.1	32.4	31.6	37.0	32.7	40.0	34.5	n.s.
The public relations by central government	32.0	27.2	35.1	34.1	34.5	43.5	34.4	n.s.
Internet	24.4	28.9	32.2	30.1	29.8	30.6	29.3	n.s.
Radio	26.2	26.6	25.7	28.3	23.4	27.1	26.2	n.s.

*χ* ^2^ test

*n.s.* not significant

*p < 0.05

There were no statistically significant differences in the perceived reliability of the central government and risk avoidance behavior regarding radiationradiation, as shown in Table 9. Overall, 21.3% of all respondents agreed that “by adhering to risk avoidance behavior as recommended by the government, citizens can adequately avoid health hazards from radiation exposure.” Overall, 54.0% of all respondents agreed that “taking into account the uncertainty of the risk assessment, citizens should avoid the risk.”

**Table 9:** Difference in the percentage of “Agree” on the reliability of central government and risk avoidance behavior regarding radiation among residents of the six prefectures

	Miyagi (*n* = 172)	Fukushima (*n* = 173)	Tokyo (*n* = 171)	Aichi (*n* = 173)	Hiroshima (*n* = 171)	Nagasaki (*n* = 170)	Total (*n* = 1030)	
Term	Agree (%)	Agree (%)	Agree (%)	Agree (%)	Agree (%)	Agree (%)	Agree (%)	Test
By performing the risk avoidance behavior according to the criteria defined by the government, citizens can avoid sufficiently health effects.	20.3	21.4	22.2	20.8	19.9	22.9	21.3	n.s.
Taking into account the uncertainty of the risk assessment, citizens should avoid the risk at your own risk	55.2	52.0	55.0	49.7	55.6	56.5	54.0	n.s.

*χ* ^2^ test

*n.s.* not significant

## Discussion

First of all, this study shows that respondents in Fukushima, followed by those in Miyagi, had an increased chance to participate in activities aimed at risk communication after the Great East Japan Earthquake, as compared to the respondents from the other four prefectures. Since participation in risk communication was entirely voluntary, this result shows that respondents in the devastated area actively sought out opportunities for participation in activities aimed at risk communication to obtain more information as well as the opportunity to appear more involved in the area.

In terms of the recognition level of the 20 technical terms related to radiation, after adjusting for demographic factors, sex, age, and residence remained significant association with “know.” On the other hand, in terms of the recognition level for the eight technical issues related to radiation, after adjusting for demographic factors, only residence remained significant association with “know.” The results indicated that careful consideration must be given more to the residence of respondents among demographic factors.

Based on the differences in the recognition level of the 20 technical terms regarding radiation among residents of the six prefectures, this study shows that respondents in Fukushima, followed by Miyagi, had an active interest in issues related to radiation health hazards. Our study shows that very familiar terms, such as WHO (85.7%) and X-ray (84.4%) were not found to be as statistically significant as the very unfamiliar term of Radiation Hazards Prevention Law in Japan. Moreover, words related to health effects of radiation such as radioactive substance, Sievert, internal radiation exposure, radioactive cesium, Becquerel, and external radiation exposure were very common in the devastated area. The basic or logistical terms, such as IAEA, radioactive strontium, natural background radiation, and half-life, were familiar to residents of Tokyo. We think this is partially attributable to higher education level among residents of Tokyo and continuing environmental contamination with radioactive substances in the Tokyo metropolitan area [5, 6]. In terms of the geographical difference, a previous study showed that students in Miyagi had higher levels of trust in institutions as compared to that among students in Tokyo [17].

In terms of the recognition level for meaning of technical issues related to radiation, this study demonstrates that appropriate information has been delivered to some extent effectively to the residents of the areas affected by the disaster and reduced their anxiety of radiation health hazards. In addition, the other second highest recognition levels for two technical issues that were “relation between radiation exposure and health effects” and “relation between radiation exposure and disease” and one technical issue of the “existence of diseases susceptible to radiation exposure” were marked by the respondents of Hiroshima and Nagasaki, respectively. This content was particularly focused on the issues of radiation exposure of human body. This implies that the residents of the A-bombed area (Hiroshima and Nagasaki) still have strong concerns related to the radiation health hazards. However, a previous study found that there was a marked bipolarization of the risk perception of the health effects of radiation among residents in Fukushima [18], and therefore, this issue should also be considered.

Regarding the topics that need to be included in forum discussions, the most interesting item was the “risk of radiation effects,” in which the uncertainty of the effects of radiation was the least interesting among the 10 items. We think that uncertainty of information causes anxiety, and to enhance public understanding, this kind of information should be delivered. In addition, the effectiveness of the food standards as countermeasures had the second highest interest level as a whole, and there were no statistically significant differences between the six prefectures in this respect. Food is directly absorbed in the body, and the facilitators should consider more scientifically sound and plain explanation to minimize the scope for misconceptions and depression [12]. Furthermore, the information needs of the respondents in this study are consistent with those reported in a previous study [19].

Regarding the sources of information on radiation and its perceived reliability, the most popular source was found to be TV, followed by the Internet and newspaper. However, regarding the reliability of the sources of information, academic journals/technical books, forums/meetings aimed at risk communication, and newspapers scored the highest. We think that since the forums/meetings for risk communication are accepted as reliable source of information, this activity should be further strengthened. However, public relations by the local and central governments occupied the fourth and fifth positions, respectively, on the hierarchy of the sources of information, but were the second lowest with respect to their perceived reliability. We think that, to a certain extent, this phenomenon may be attributable to the more persuasive attitude of the central government towards the citizens.

Overall, approximately one fifth of respondents agreed that “by adhering to risk avoidance behavior according to the criteria defined by the government, citizens can adequately prevent adverse health effects.” The majority of respondents also agreed that “taking into account the uncertainty of the risk assessment, citizens should avoid health risks.” The central government should consider these results sincerely to improve the efficacy of risk communication since trust is one of the critical pre-requisite for risk communication [20].

Because answers can be obtained in a short time from monitors previously registered with an Internet research company, Internet surveys have been accepted in the field of medical sociology in Japan [14–16]. However, face-to-face interviews are considered more reliable for assessment of the degree of the recognition level of technical terms. Therefore, we consider this to be one of the limitations of this study. In addition, the population in each prefecture varies substantially but the sample size of 160 was set based on the results of the similar researches [14–16]. This also should be considered as another limitation of this study.

## Conclusion

We confirmed that the respondents in the Tohoku region had a better recognition for the technical terminology relevant to radiation health hazards. We recommend continuation and reinforcement of activities aimed at risk communication related to radiation health hazards, by central/local governments and other stakeholders, to reduce anxiety among the residents of Tohoku region.

## Abbreviations

IAEA: International Atomic Energy Agency
ICRP: International Commission on Radiological Protection
OR: Odds ratio
WHO: World Health Organization
